# Volatility of Mutator Phenotypes at Single Cell Resolution

**DOI:** 10.1371/journal.pgen.1005151

**Published:** 2015-04-13

**Authors:** Scott R. Kennedy, Eric M. Schultz, Thomas M. Chappell, Brendan Kohrn, Gary M. Knowels, Alan J. Herr

**Affiliations:** 1 Department of Pathology, University of Washington, Seattle, Washington, United States of America; 2 Department of Entomology, North Carolina State University, Raleigh, North Carolina, United States of America; Université Paris Descartes, INSERM U1001, France

## Abstract

Mutator phenotypes accelerate the evolutionary process of neoplastic transformation. Historically, the measurement of mutation rates has relied on scoring the occurrence of rare mutations in target genes in large populations of cells. Averaging mutation rates over large cell populations assumes that new mutations arise at a constant rate during each cell division. If the mutation rate is not constant, an expanding mutator population may contain subclones with widely divergent rates of evolution. Here, we report mutation rate measurements of individual cell divisions of mutator yeast deficient in DNA polymerase ε proofreading and base-base mismatch repair. Our data are best fit by a model in which cells can assume one of two distinct mutator states, with mutation rates that differ by an order of magnitude. In error-prone cell divisions, mutations occurred on the same chromosome more frequently than expected by chance, often in DNA with similar predicted replication timing, consistent with a spatiotemporal dimension to the hypermutator state. Mapping of mutations onto predicted replicons revealed that mutations were enriched in the first half of the replicon as well as near termination zones. Taken together, our findings show that individual genome replication events exhibit an unexpected volatility that may deepen our understanding of the evolution of mutator-driven malignancies.

## Author Summary

Mutations fuel microbial evolution and cancer. Cells with an increased rate of mutation are said to have a “mutator phenotype” and adapt more rapidly than non-mutator cells. Our study utilizes a novel way of measuring mutation rates of individual cell divisions to show that mutator cells can adopt one of two mutation rates that differ tenfold in magnitude. This mutator volatility suggests that the rates of mutation accumulation may vary widely within the same clone of mutator cells. Understanding how to modulate the mutator state may provide an avenue to treat certain cancers.

## Introduction

A network of DNA metabolic activities maintains genomic integrity during each cell division [1], ensuring that eukaryotic mutation rates remain less than one mutation per billion base-pairs synthesized. Defects to these activities can lead to mutator phenotypes that increase the rate of mutation [2]. As the mutator population expands, genetic diversity increases, fueling evolution. In multi-cellular organisms, mutator phenotypes accelerate tumorigenesis by generating mutations that overcome the genetic and environmental barriers to unrestrained proliferation [3,4]. In tumors that are not initially mutator-driven, chemotherapeutic treatment provides selection pressure for sub-clonal mutator cell lineages to emerge, which more easily evolve drug-resistance. Thus, mutator phenotypes may pose substantial challenges to cancer therapy, necessitating a greater understanding of their inherent vulnerabilities.

The most abundant source of potential mutations in dividing cells are polymerase errors, which are corrected by the synergistic activities of polymerase proofreading and mismatch repair (MMR) [2]. Pol ε and Pol δ perform the bulk of leading and lagging strand DNA replication in eukaryotes, respectively [5], and contain intrinsic proofreading exonucleases that excise the vast majority of polymerase errors. Mismatches that escape proofreading are recognized by Msh2•Msh6 (base-base mismatches, insertion/deletion mispairs) or Msh2•Msh3 (primarily insertion/deletion mispairs) [2]. These complexes recruit the endonucleases Mlh1•Pms1(Pms2 in mammals) or Mlh1•Mlh3, which initiate processing and re-synthesis of the DNA [2]. Defects to proofreading or MMR increase mutation rates in microbes and mammalian cells [2]. In humans, mutations that compromise Pol ε or Pol δ proofreading or MMR lead to colorectal (CRC) and endometrial cancers (EC) [6–11], supporting the hypothesis that maintenance of DNA replication fidelity restrains neoplasia [3,4,12–14]. Synergistic defects in both MMR and proofreading greatly accelerate tumorigenesis [15]. Since proofreading and MMR act in series on the same pool of errors, concomitant defects in these activities elevate mutation rates 10,000-fold in diploid yeast [16–18]. In haploid yeast, this level of mutagenesis causes error-induced extinction [16,17,19,20]. Not all proofreading and MMR defects are synthetically lethal to haploids. Yeast cells with mutant alleles for Pol ε proofreading deficiency (*pol2-4*) and Msh6 (*msh6Δ*) exhibit mutation rates of only 1000-fold greater than background [21,22], just below the critical level at which haploid colony forming capacity declines [20].

Strong mutator phenotypes may be more volatile than commonly appreciated. The first hints of hypermutability came from differences observed between haploid and diploid yeast in the rates of base-analogue 6-hydroxylaminopurine (HAP) induced mutagenesis [23,24]. Subsequent studies revealed a wide variability in the mutagenesis induced in diploids by HAP or AID/APOBEC cytosine deaminase expression: clones selected for a mutant phenotype had much higher genome-wide mutational loads than unselected clones exposed to the same mutagenic treatment [25]. A similar hypermutable state has been advanced to explain why diploid strains deficient in Pol δ proofreading display mutation rates 3 to 20-fold greater than isogenic haploid yeast strains [26]. These results are consistent with the hypothesis that some Pol δ proofreading-deficient cells enter a “hypermutator” state, which is lethal to haploids but tolerated by diploids [26]. If mutational processes are similarly volatile during tumorigenesis, they may influence the rate of tumor evolution and the nature of genetic diversity present in the growing tumor clone.

Testing for mutator volatility has proven technically challenging. Historically, mutator phenotypes have been measured by scoring the frequency of rare mutations in selectable genes in thousands or even millions of cells. Analysis of the fluctuation in mutation frequencies in multiple independent cultures yields the mutation rate of the target gene during clonal expansion [27]. In an alternative approach, individual cell lines are propagated through bottlenecks over hundreds or thousands of generations and then analyzed by whole genome sequencing to derive the generational mutation rate [28,29]. Both of these methods can only report the average mutation rate of the entire population, which obscures the actual mutation rate for any given replication event. Overcoming this limitation requires the measurement of mutation rates at single cell resolution. As an experimental approach, single cell DNA sequencing holds promise, but current methods require *in vitro* enzymatic amplification of the genome [30,31]. Because DNA polymerases are used to amplify the DNA, base misincorporation events can lead to the scoring of thousands of false mutations. Additionally, the spatial and temporal relationship between cells is lost in these experiments; thus, it is impossible to know precisely how many cell divisions occurred between any two related cells. We devised an alternative approach, which is to sequence clones of cells derived from sequential cell divisions of the same cell lineage. Each clone contains the mutational history of the replication event, as well as all previous genome replications. By comparing clones derived from sequential cell divisions, it is possible to determine precisely when each mutation arose. Here, we apply this strategy to determine the fidelity of individual genome replication events of *pol2-4 msh60Δ* mutator yeast cells.

## Results

**Fig 1:**
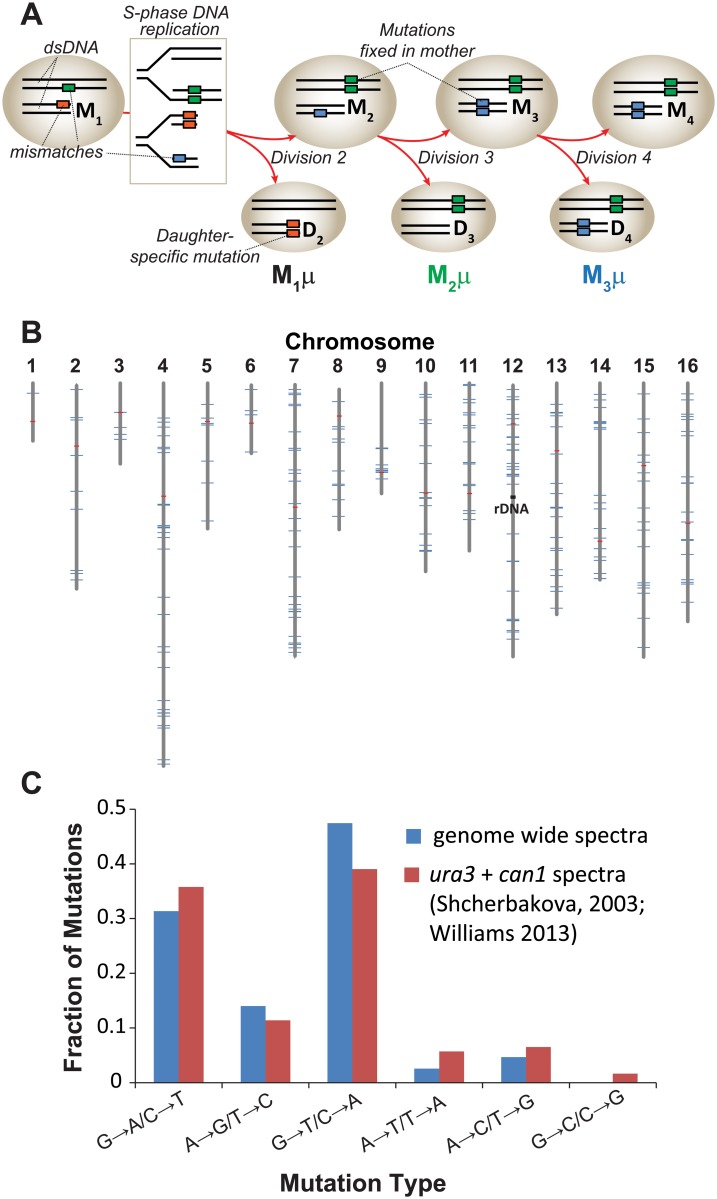
Mutation rate of *pol2-4 msh6Δ* mother yeast cells at single cell resolution. (A) Polymerase errors (orange, green, and blue boxes) arising in maternal double-stranded DNA (dsDNA) as mismatches become mutations during S-phase DNA replication (see rectangle) and segregate to the mother (M) or daughter (D) cells. Subscript numbers following M or D indicate the division number that produced the cell (e.g. M_1_ is the mother cell after one division). Red arrows indicate only one of several segregation scenarios. Single cell mutation rates (*M*_*1*_ *μ*, *M*_*2*_ *μ*, *M*_*3*_ *μ*) are defined as the number of new mutations fixed in the maternal lineage at each cell division divided by the total number of nucleotides sequenced in all members of a lineage. (B) Genomic distribution of the 237 mutations observed in individual cell divisions (blue lines) among the 16 yeast chromosomes (gray lines). Red lines, centromeres. (C) Mutation spectra of *pol2-4 msh6Δ* cells from whole genome sequencing (blue) compared to published spectra (red).

We used budding yeast cells to investigate mutation rates of individual cell divisions because they divide asymmetrically into “mother” and “daughter” cells that are easily separated by micromanipulation. The daughter cells readily expand into clones, which can then be subjected to whole genome sequencing to ascertain mutations. During the first division of the mother cell (M_0_→M_1_), new DNA replication errors retained by the mother (M_1_) in the form of mismatches become mutations in the next round of replication and segregate to the daughter (D_2_) or are retained by the mother (M_2_) (Fig 1A). Mutations and mismatches segregated to the daughter will be unique to her clone of cells, whereas mutations retained by the mother cell (maternal mutations) will appear in the third daughter (D_3_) clone and all subsequent daughter clones (e.g. D_4_, D_5_, etc). The number of new maternal mutations in each daughter clone can be used to determine the mutation rates of individual maternal cell divisions.

As a source of mother cells, we used haploid spores freshly dissected from tetrads derived from meiosis of a diploid strain that was heterozygous for both alleles (*POL2/URA3*::*pol2-4 MSH6/msh6Δ*::*LEU2*). The four haploid genotypes from this strain are: 1) wild-type (WT) with respect to replication fidelity (*POL2 MSH6*), 2) proofreading defective (*pol2-4 MSH6*), 3) MMR defective (*POL2 msh6Δ*), or 4) proofreading and MMR defective (*pol2-4 msh6Δ*). Canavanine-resistance (Can^r^) mutation rates determined by fluctuation analysis [32–34] revealed that the *pol2-4 and msh6Δ* alleles individually increased mutation rates 3 and 10 times above background, respectively (S1A Fig) [21,22]. The *pol2-4 msh6Δ* cells have mutation rates of 1.7 x 10^-4^ Can^r^ mutants per cell division (S1A Fig), which corresponds to 4.3 x 10^-7^ mutations/bp/cell division using the method of Drake [35] that we employed previously [20]. Assuming at least 80% sequencing coverage of the haploid yeast genome (1.2 x 10^7^ bp) and that the mutability of CAN1 is representative of other genes, we estimated we would observe an average of 4 to 5 mutations for each division of pol2-4 msh6Δ cells [(4.3 x 10–7 mutations/bp/cell division) x (1.2 x 107 bp) x 0.8 = 4 mutations].

To establish single cell lineages, we randomly chose spores to serve as mother cells and moved them to a defined location on an agar plate. When the mother cells began dividing, we moved all daughters to unique positions on the plate where they formed colonies (S1B Fig). We then sequenced the genomes of the viable daughter clones, scoring only mutations at genomic positions accurately called in all members of a lineage. In all lineages, we assessed greater than 80% of the yeast genome (1.05 (±0.05) x 10^7^ base-pairs; Table 1). We then compared the sequence of the last viable daughter clone to the sequences of all earlier daughter clones to define mutations fixed in the maternal lineage at each cell division. We observed no mutations in three WT control lineages, while two mutations were fixed during 9 cell divisions of an *msh6Δ* mother cell (Lineage A in S1 Dataset). In contrast, we observed an average of 30±7 mutations during 11±3 divisions of *pol2-4 msh6Δ* mother cells (Lineages B-H in S1 Dataset, summarized in S1 Table). All told, 237 mutations accumulated over 87 divisions of *pol2-4 msh6Δ* cells (Table 1). The average mutation rate was 2.6 x 10^-7^ mutations/bp/cell division, determined by dividing the total number of mutations in all lineages by the total base-pairs scored (Table 1). The mutations were distributed across all 16 chromosomes (Fig 1B), although mutation rates for individual chromosomes varied six-fold (S1C Fig). The mutation spectra, characterized by high numbers of GC→AT and GC→TA mutations, corresponded well with the combined published *ura3* and *can1* mutation spectra of *pol2-4 msh6* cells (Fig 1C, S2 Table), as well as with spectra obtained with purified proofreading-deficient Pol ε [21,22]. AT→TA mutations, also frequently observed *in vitro*, were relatively less abundant in the whole genome spectra, but this can be explained by the preferential repair of these mismatches by the Msh2•Msh3 complex [36], which remains active in *pol2-4 msh6Δ* cells.

**Table 1:** Genome sizes and mutation rates of *pol2-4 msh6*Δ sequenced lineages.

*Lineages* ^*a*^	*Sequenced Genome Size* ^*b*^	*Mutations*	*Divisions*	*Mutation Rate* ^*c*^	*Total Base-Pairs Scored* ^*d*^
*SC media*					
C	10034503	41	13	3.1	130448539
F	11040018	31	12	2.3	132480216
G1	9684038	32	11	3.0	106524418
G2	10209723	18	5	3.5	51048615
H	10826564	30	12	2.3	129918768
*SC-Ura-Leu*					
B	10926041	19	9	1.9	98334369
D	10099122	36	15	2.4	151486830
E	10889177	30	10	2.8	108891770

*Sum*		237	87		909133525

*average*	1.02E+07	30	11	2.7	*average mutation rate* ^*e*^
					
*stdev ±*	4.98E+05	7	3	0.5	2.6

^a^Lineages C, F, G1, G2, and H were grown on synthetic complete (SC) media. Lineages B, D, and E were grown on synthetic complete media without uracil and leucine (SC-Ura-Leu) to select for double mutant spores containing the *URA3* and *LEU2* transgenes linked to the mutator alleles.

^b^The sequenced genome size equals the number of base-pairs of the genome confidently scored in all members of a lineage.

^c^Values below this heading should be multiplied by 10^-7^ mutations/base-pair/cell division.

^d^Obtained by multiplying the sequenced genome size by the number of cell divisions scored.

^e^The value below this heading was determined by dividing the total number of mutations by the number of cell divisions and the total base-pairs confidently scored. It should be multiplied by 10^-7^ mutations/base-pair/cell division.

**Fig 2:**
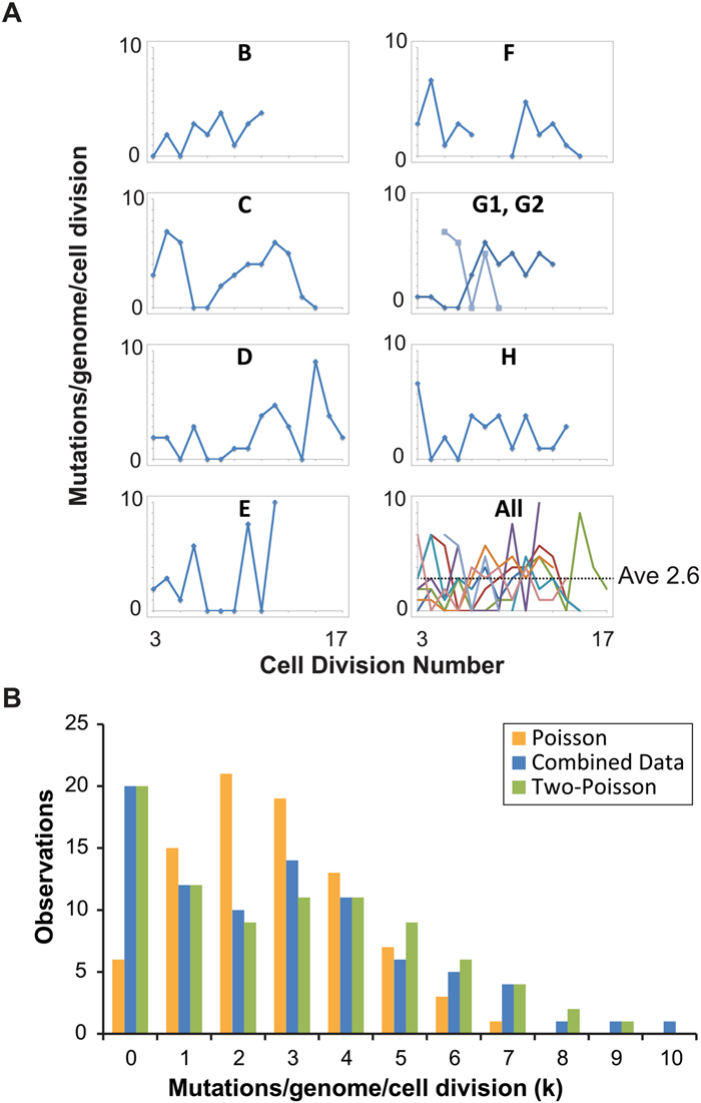
Distribution of single cell mutation counts in mother cells. (A) Mutation counts of mutator cells during yeast aging. Each lineage is plotted separately, except Lineage G, whose mother cell became multi-budded, producing two distinct lineages: G1 (blue) and G2 (light blue). The gap in Lineage F is due to sequencing failure of one of the daughter clones. *Bottom right*, all lineages are plotted together, each represented by a different line color. (B) The observed distribution (blue bars, combined data) of mutation counts is plotted against the predicted Poisson distribution based on the average genome-wide mutation rate (2.6 x 10^-7^ mutations/bp/cell division) (orange bars, Poisson Model) and a composite distribution resulting from two overlapping Poisson distributions with mutation rates of 4 x 10^-8^ (contributing 35%) and 4 x 10^-7^ (contributing 65%) mutations/bp/cell division (green bars, Two-Poisson Model).

Individual cell divisions exhibited considerable variation in mutation counts (Fig 2A) that did not correlate with the replicative age of the mother cell (r = 0.1, p = 0.4, Pearson). We modeled mutagenesis in *pol2-4 msh6Δ* cells as a Poisson process to test whether this variation could be explained by a single overall mutation rate. Long used to model random mutagenesis [27,37], the Poisson function calculates the probability of a defined number of rare events (*k) occurring within a fixed interval of time at a given rate parameter (λ)*. In Eq 1, P_k_ is the probability of cells fixing *k* mutations in a single cell division. For our purposes, *λ* is defined as μ, the average per-base-pair mutation rate (2.6 x 10^-7^ mutations/bp/cell division from Table 1), times G, the size of the sequenced genome in base pairs; *e* is the base of the natural logarithm.

$$p_{k}\;=\;\frac{{\left(\mu G\right)}^{k}\;e^{-(\mu G)}}{k!}$$ (1)

The resulting probability for each value k, multiplied by the number of cell divisions scored, gives the expected number of divisions with k mutations. We separately modeled Poisson distributions to the data of each lineage to account for differences in the number of base-pairs sequenced and cell divisions scored (S2A Fig) and then summed the Poisson distributions together. The resulting “Summed-Poisson model” poorly fit the combined observations from all lineages due to overdispersion of the data (Fig 2B, S2B Fig). We considered the possibility that overdispersion could be due to zero-inflation from under-sampling of genomic sites in lineages with the smallest sequenced genome sizes (Lineages C, D, G1, G2, Table 1). However, we found no correlation between the number of samples in a lineage with 0 mutations and the size of the sequenced genome, suggesting that under-sampling is not the source of the overdispersion (S3 Fig).

We reasoned that overdispersion could result from a mixture of underlying distributions generated by two or more mutation rates. To test this hypothesis, we first generated a simplified model of the data that grouped all cell divisions into one set, utilizing the average genome size (1.02 x 10^7^ bp, Table 1). The Simplified and Summed Poisson distributions were virtually identical (S2C Fig), suggesting that the data could indeed be modeled as a single set. To compare how well single-distribution and distribution-mixture models fit the data, we used finite mixture modeling (FMM), which is a computational approach that fits mixtures of parametric distributions to data [38]. Because the Poisson distribution is described by the single parameter λ (μG in eq 1), the number of parameters in each model is equal to the number of Poisson distributions underlying the composite probability mass function. Fitted models included one to five parameters (i.e., mutator states). Because parameter dimensionality increases by one between each of the five fitted models, a best-fit model was selected by comparison using maximum-likelihood-ratio tests of nested hypotheses with one degree of freedom for each test. The best-fit model described the data significantly better than models with fewer parameters, and not significantly worse than models with more parameters. The best fit model by maximum likelihood estimation was a Two-Poisson distribution with values for λ of 0.402 and 3.897. The difference between the Poisson and Two-Poisson distributions by the likelihood ratio test was highly significant (Likelihood Ratio, Chi-Square Test = 40.58, Degrees of Freedom = 1, p < 1.9 x 10–10). The difference between the best Two-Poisson and Three-Poisson models was insignificant (Likelihood Ratio, Chi-Square Test = 0.80, Degrees of Freedom = 1, p = 0.37), indicating that increasing the number of Poisson distributions in the mixture does not improve the fit to the data. Thus, the best-fit model was a mixture of two Poisson distributions.

To study the relative contribution of these two distributions to the observed mutation count, we constructed a “Summed Two-Poisson” model that took into account differences between the lineages (S2 Dataset). We calculated the expected single Poisson distributions of mutation counts for each lineage assuming a hypo- (0.4 x 10^-7^ mutations/bp/cell division) or hypermutator state (4 x 10^-7^ mutations/bp/cell division). We summed the Poisson distributions from all lineages to obtain the expected distribution of mutation counts across the entire experiment for cells with a hypo- or hypermutator state. We then combined the hypo- and hypermutator Poisson distributions, with each contributing 50% to the final mixture. After comparing this mixture to the observed distribution of the data, we adjusted the contribution of each mixture component, ultimately finding that a model with 35% hypomutator divisions and 65% hypermutator divisions provided the best fit (Fig 2B). Thus, on the strength of the above hypothesis testing and modeling, we propose *pol2-4 msh6Δ* mutator mother cells assume either a hypomutator state or a hypermutator state as they pass through S-phase, with mutation rates that differ by an order of magnitude.

**Fig 3:**
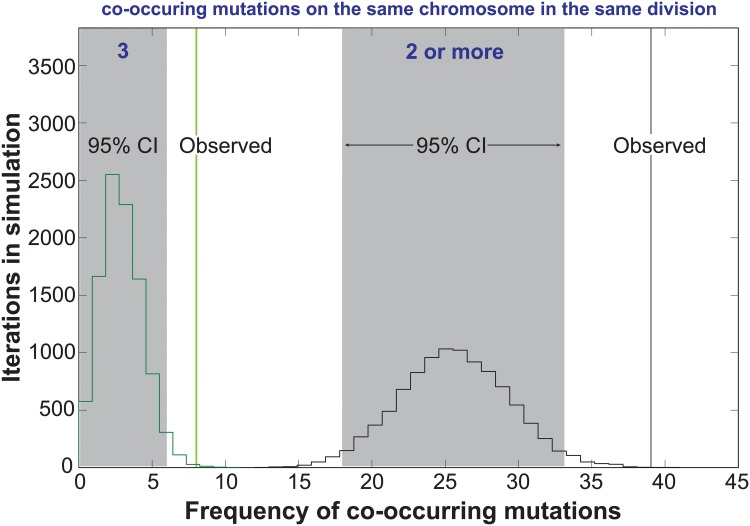
Co-occurrences of mutations in the same chromosome and cell division. The random accumulation of 237 mutations over 87 cell divisions was simulated using 10,000 iterations. green histogram, co-occurrences in the simulation with 3 mutations on the same chromosome; green vertical line, actual co-occurrences with 3 mutations; black histogram, co-occurrences with 2 or more mutations on the same chromosome; gray shading, 95% confidence intervals; black vertical line, actual co-occurrences with 2 or more mutations.

In the Two-Poisson model, the bulk of observed mutations would arise in cell divisions with a hypermutator state. Only 13 of the 237 mutations would arise during hypomutator cell divisions. Close examination of the mutations in error prone cell divisions revealed numerous instances in which pairs or trios of mutations occurred on the same chromosome. To determine the significance of this pattern, we computationally simulated the experiment 10,000 times, assuming random mutagenesis. Each round of the simulation returned a value for the number of random co-occurrences of 2 or 3 mutations on the same chromosome. Plotting the values from all 10,000 iterations gave 95% confidence intervals of 18 to 33 observations of 2 or more mutations on the same chromosome in the same cell division and 0 to 6 observations of 3 mutations. We observed 39 instances of 2 or more mutations and 8 observations of 3 or more mutations (Fig 3), suggesting that the hypermutator state may be expressed in only a portion of the genome in a given cell cycle.

To explore the relationship between mutagenesis and replication dynamics, we mapped mutations onto the yeast DNA replication profiles from Raghuraman and colleagues [39] (S4 Fig). These replication profiles, generated using isotopic labeling time-course experiments and high density microarrays, report the timing (t_rep_) of 50% DNA replication within a sliding 10kb window, quantified every 500 base-pairs across the genome. Local maxima and minima represent putative locations for origins and termination zones and the line between the two denotes replicons. We found the chromosomal positions of mutation pairs occurring in the same chromosome and cell division were not correlated (r = 0.24, p = 0.2, S5A Fig, S3 Dataset). Only five sets of co-occurring mutations reside in the same replicon (S4 Fig, see Chrs. 4, 12, 13, and 16), with two pairs affecting the same replicon on chromosome 12 (see purple and teal triangles). The remaining co-occurring mutation pairs were separated by multiple replicons, consistent with the hypothesis that they arose from independent Pol ε complexes.

Intriguingly, co-occurring mutation pairs frequently reside in DNA on the same chromosome with similar t_rep_ values (r = 0.47, p = 0.006, 2-tailed, Pearson, S5B Fig, S3 Dataset) [39], suggesting that the polymerase errors may have been committed at a similar time during S-phase. The same correlation is not apparent when all pairwise combinations of co-occurring mutation trios are considered; however, in 6 out of 8 trios, two of the three mutations did occur in DNA with similar t_rep_ values, consistent with a temporal relationship. We also examined the predicted replication timing of mutations occurring on different chromosomes in the same cell division. As with the mutation trios, no correlation is observed when all pair-wise combinations are considered. These observations suggest that while the hypermutator state may have periods of increased mutagenesis affecting a fraction of the genome, it may not be temporally constrained.

**Fig 4:**
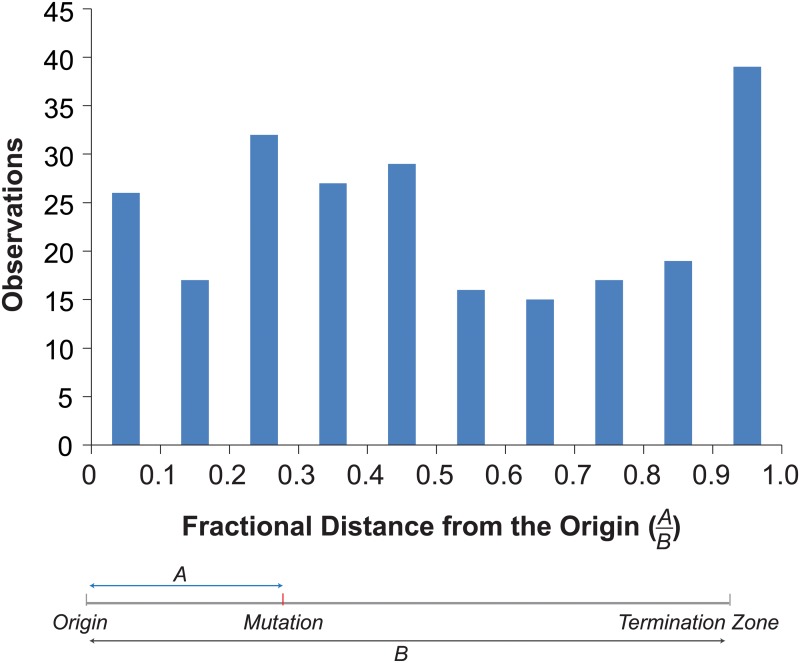
Distribution of mutations within replicons. *Top*. Histogram of the fractional distances of mutations to the nearest origin using the replicons defined by Raghuraman et al. [39] (see S3 Dataset). *Bottom*. Schematic shows how fractional distances are determined.

Mapping the mutations onto the replication profiles revealed an enrichment of mutations near origins and termination zones (Fig 4). To examine the overall distribution of mutations within replicons, we determined the distance of each mutation to the nearest origin and termination zone [39], and binned mutations by their fractional distance to the origin in 0.1 increments (Fig 4). More mutations occurred in bins closer to the origin (130) than in bins closer to the termination zone (107). The disparity would have been even greater were it not for a dramatic increase in the number of mutations in the bin closest to the termination zone (Fig 4). This distribution of mutations significantly deviated from that expected by chance (Chi-Square Test, p = 0.005), suggesting that mutagenesis in *pol2-4 msh6Δ* cells may be influenced by the dynamics of replication.

## Discussion

Our work demonstrates that strong mutator phenotypes can be studied at single cell resolution through whole genome sequencing of sequential daughter cell clones. This strategy could in principle be adapted to any cell type amenable to single cell cloning. In yeast this approach is currently limited to strains with very high mutation rates. Expanding this method to organisms with much larger genomes, while not currently feasible, would permit the measurement of mutation rates much lower than what we describe here. Fluctuation analyses and mutation accumulation lines will continue to be valuable tools for the study of mutagenesis; however, our new approach reveals what these classic techniques cannot—whether mutator phenotypes are constant in every cell division. The *pol2-4 msh6Δ* cells we analyzed exhibited a strong mutator phenotype and mutation spectrum consistent with high levels of unrepaired Pol ε errors (Fig 1C, S1A Fig). Since the mutations in pol2-4 msh6Δ cells depend on the synergy between *pol2-4* and *msh6Δ* (S1A Fig) they almost certainly derive from Pol ε replication errors during leading strand DNA synthesis. The mutation accumulation observed in individual cell divisions, as well as our subsequent modeling, indicates that individual *pol2-4 msh6Δ* cells can exhibit both hypo- and hypermutator states (Fig 2B). These mutator states appear to be transient, as some mother cells exhibited alternatingly low and high mutation rates in consecutive cell divisions. The idea of multiple mutator states is supported by the surprising finding that mutations co-occur on the same chromosome more frequently than expected by chance (Fig 3). The mutation pairs usually reside in different replicons and often in genomic regions with similar replication timing, suggesting that the hypermutator state may alter the fidelity of distinct replication complexes copying the same chromosome at the same time (S4 Fig and S5B Fig). The predicted replication timing of mutations arising on other chromosomes in the same cell division is not correlated, suggesting that the hypermutator state is not temporally constrained. Finally, examination of the locations of mutations within predicted replicons reveals an unexpected increase in mutation frequency near termination zones (Fig 4). In what follows, we discuss the hypothesis of multiple mutator states and alternative models, the connection between replication dynamics and mutagenesis, and the implications of our findings for understanding the evolution of mutator populations in cancer.

### Multiple mutator states and alternative models

Mutagenesis has long been modeled as a Poisson process under the assumption that mutations occur independently in each cell division with a constant mutation rate parameter [27,37]. We set out to test the hypothesis that mutation accumulation in pol2-4 msh6Δ cells results from a single Poisson process guided by a constant mutation rate. Using finite mixture modeling and likelihood ratio tests we found that a Two-Poisson model fit the mutation count data significantly better than a single Poisson model. Thus, a single mutation rate does not appear to underlie the generation of mutations in pol2-4 msh6Δ cells. As an alternative to the simple idea of two mutator states, we also considered a negative binomial model in which each cell division adopts a different mutation rate, with the rates being gamma distributed. This scenario produces a Poisson model. We compared the negative binomial and Two-Poisson models using Akaike’s Information Criterion (AIC) [40], which distinguishes between classes of models on the basis of both goodness-of-fit and parsimony. The AIC for the negative binomial model is 368.49, whereas the AIC for the Two-Poisson model is 363.93 (lower is better). The relative likelihood ratio of the negative binomial model over the Two-Poisson model is 0.102, indicating that the Two-Poisson model provides a better fit to the data.

In the Two-Poisson model, we have proposed that the distribution of mutation counts is caused by two underlying mutation rates. However, a second statistical process determines how many new mutations appear in a mother cell besides mutation rate: namely the segregation of mutations between mother and daughter cells. It is conceivable that there is a single underlying mutation rate, but two types of biased segregation patterns. In one segregation pattern, the mother cell would retain the mutations with a high probability. In the other pattern, the mother would have a low probability of retaining the mutations. By varying the frequency of the two segregation patterns and the degree of segregation bias, it is possible to produce two overlapping Poisson distributions identical to the two-state mutator model. While we cannot formally distinguish between the two models, we find it easier to imagine how the error rates of mutator polymerases may be modulated than how there would be two distinct unequal segregation patterns of mutations.

The distinct mutator states proposed for pol2-4 msh6Δ cells could derive from a process that influences the mutator activity of proofreading-defective Pol ε. The modeled per-genome mutation rate of the hypomutator state (0.4) matches the rates recently described for MMR-deficient haploid (0.71) and diploid (0.38) yeast mutation accumulation lines [41]. This correspondence suggests that cell divisions with a hypomutator state do not appreciably express the Pol ε proofreading-deficient phenotype. There may be a regulatory switch that influences Pol ε rates of misincorporation or mispair extension, the action of alternative repair mechanisms that edit Pol ε errors, or the extent to which proofreading-deficient Pol ε contributes to the overall replication of the genome.

Our proposal for two distinct mutation rates in mutator cells contrasts previous work with Escherichia coli, which found that mutation count data in individual mutator cells conforms to a single Poisson distribution [42]. This work elegantly followed the occurrence of likely mutations by the formation of persistent, fluorescently labeled MMR foci that form when there is a failure to repair a mismatch. The contrasting results between the two studies could stem from either technical or biological differences between the two experimental systems. A notable limitation of counting fluorescent foci is the potential for undercounting. Specifically, it may be difficult to resolve high numbers of foci in cells with a hypermutator state, especially if the mutations occur in close proximity. In addition, if the hyper-mutator state saturates MMR, not all mismatches would lead to fluorescent foci. Genetically, we relied on tandem deficiencies in MMR and polymerase proofreading to raise mutation rates to an appreciable level, whereas the work in bacteria used strains deficient in either MMR (*mutH*) or proofreading. Of course, intrinsic differences between prokaryotic and eukaryotic DNA replication and repair could also explain the contrasting data sets. In particular, prokaryotes utilize a single replicative polymerase (Pol III) for both leading and lagging strand bulk DNA synthesis whereas eukaryotes divide the labor between Pol δ and Pol ε. As discussed below, Pol δ may replace Pol ε at some point during leading strand replication, providing a potential avenue by which the contribution of proofreading-deficient Pol ε to genome replication and mutagenesis may be modulated.

### Replication dynamics and mutagenesis

An important clue to the mutator volatility of pol2-4 msh6Δ cells lies in the observation that pairs of mutations occurred on the same chromosome more frequently than expected by chance (Fig 3). The correlation in predicted replication timing of co-occurring mutation pairs suggests that the hypermutator state may be linked to replication dynamics of individual chromosomes. Chromosomes occupy distinct regions within the nucleus [43] and evidence exists for replication factories consisting of multiple active replisomes acting on distinct origins with similar replication timing [44]. Thus, changes in replication fidelity could be factory-specific, restricting the mutator phenotype to limited regions of the genome in a given cell cycle. If the phenomenon that underlies focal expression of the hypermutator state were to extend to the entire genome, much higher genome-wide mutation rates may result. Our ability to detect much higher genome-wide mutation rates in the current study is limited by the likelihood that extreme mutator states would result in haploid lethality. Future studies with diploid cells, which are buffered against recessive deleterious mutation accumulation, will be required to explore the full extent of mutator volatility.

We investigated the link between replication dynamics and mutagenesis by mapping mutations onto published replication profiles [39]. This approach has some limitations worth noting. Replication profiles average the replication timing of large numbers of cells, yet replication initiation events are probabilistic phenomena that vary between cells [45]. Variation in origin firing likely leads to variation in the termination of DNA replication as most termination occurs independently of sequence context within zones located between adjacent firing origins [46]. Since no two cells follow the same temporal order of origin firing, there may be substantial variation in replicons. Someday it may be possible to monitor replication fidelity and dynamics simultaneously. Until then, we feel the best approach is to map mutations onto replicons that incorporate the probability of origin firing, recognizing that in some cases, these “probabilistic” replicons may differ from the actual replicons in which the mutations occurred. In our study, we normalized the positions of mutations within the probabilistic replicons using fractional distances (Fig 4). In cases where the probabilistic and actual replicons differ, the fractional distances would be inaccurate and likely diminish any signal for the enrichment of mutations near termination zones. In our view, this makes evidence for enrichment even more compelling.

Ample evidence supports the hypothesis that Pol ε performs leading strand DNA replication near origins of replication [5,47]. Whether Pol ε remains the leading strand polymerase through the end of each replicon continues to be debated [48]. Replacement of Pol ε with Pol δ may be important for joining the leading strand with the downstream Okazaki fragment [49]. We found more mutations in the first half of the replicon than in the second half. This uneven distribution is consistent with a model in which proofreading-deficient Pol ε is replaced by Pol δ with increasing probability as replication proceeds [48]. If replacement of Pol ε with Pol δ after initiation is subject to regulation, the hypomutator state could be explained by hyper-activation of a mechanism that replaces proofreading-deficient Pol ε with Pol δ. We also found an unexpected concentration of mutations near the termination zones, suggesting that proofreading-deficient Pol ε replisomes that do make it to the termination zone may become especially error prone.

The relationship between mutagenesis and replication dynamics has also been explored in a recent mutation accumulation study that utilized MMR-deficient strains expressing either WT Pol ε or a mutant variant with the M644G substitution in the polymerase active site [50]. In this case, replicons were defined as the distance between origins and the inter-origin midpoints, which serve as proxies for termination zones [50]. Mutation frequency was constant across the defined replicons for Pol ε-M644G-expressing MMR-deficient cells, but increased significantly near the inter-origin midpoints in WT Pol ε MMR-deficient controls.

The nature of each Pol ε variant may account for these distinct patterns of mutagenesis. Pol ε-M644G retains proofreading activity, but elevates the rates of both misinsertion and mispair extension [47]. Whereas polymerase pausing at mispaired primer termini may trigger the replacement of proofreading-deficient Pol ε with Pol δ, the propensity of Pol ε-M644G to extend mispairs may limit this exchange, ensuring mutagenesis extends to the end of the replicon. The enrichment of mutations near termination zones in MMR-deficient cells with either WT or proofreading-deficient Pol ε suggests that replication fork convergence is an error-prone process, monitored by MMR. The absence of a signal in the Pol ε-M644G data may indicate Pol ε-M644G does not become any more error-prone near termination zones. However, a subtle but significant enrichment of mutations near termination zones may lie hidden within the Pol ε-M644G data—probabilistic differences in the firing of adjacent origins mean that termination zones are not always at the inter-origin midpoint. Consistent with this, when we analyze our data using inter-origin midpoints rather than the defined termination zones, we find only a muted enrichment of mutations near the midpoint (S6 Fig).

### Implications of mutator volatility

The ability of mutator cells to assume distinct mutagenic states may have important implications for understanding the remarkable mutation accumulation observed in tumors with Pol ε proofreading defects [7–11,51]. *POLE* tumors are often microsatellite-stable, suggesting that the high mutation burden of *POLE* cells does not simply result from synergy between proofreading and MMR defects [7,51] The *POLE* cancer alleles are usually heterozygous in the tumor clones [51]. Modeling of the most common allele (*POLE-P286R*) in diploid yeast (*pol2-P301R*) reveals a strong semi-dominant mutator phenotype [52] that contrasts the weak semi-dominant mutator phenotype of *pol2-4* (*pol2-D290A*, *E292A*) [52]. It is conceivable that if the *POLE* cancer alleles confer volatile mutator phenotypes, the spread between the highest and lowest mutator states may be much larger than we observed with *pol2-4*. *POLE* cells that pass through a hypermutator state would acquire adaptive mutations more readily. Over multiple rounds of selection during tumor evolution this would lead to an extremely rapid accumulation of mutations within the dominant tumor clone. The average mutation frequency in the exomes of *POLE* tumors (235 x 10^-6^ mutations/bp) [11] appear to be near the lethal limit for diploids [15,53]. Thus, once a *POLE* tumor clone escapes the restraints on growth, there may be strong selection pressure to limit mutation accumulation, giving an advantage to cells in a hypo-mutator state. Single cell resolution replication studies of human *POLE* mutant cells are needed to test the hypothesis of mutator volatility in cancer. Understanding the source of volatility may lead to treatments that directly target the mutator phenotype for cancer therapy.

## Materials and Methods

### Media and growth conditions

Yeast were grown at 30°C using YPD, synthetic complete (SC) media or SC “drop-out” media deficient in defined amino acids to select for prototrophic genetic markers [54]. Premade nutrient supplements for SC and SC lacking uracil (SC-Ura) and leucine (SC-Leu) were purchased from Bufferad. Other drop-out nutrient supplements were made as described [54] from individual components purchased from Sigma-Aldrich or Fisher Scientific. Canavanine-resistant (Can^r^) mutants for mutation rate assays were selected on SC plates lacking arginine that contained 60 μg/ml canavanine (Sigma-Aldrich).

### Strain construction

To construct AH2801, the *POL2/URA3*::*pol2-4 MSH6/msh6Δ*::*LEU2* diploid used in these experiments, we first deleted one of the two copies of *MSH6* in AH0401 [53] to obtain AH0604. We transformed [55] AH0401 with a *LEU2* DNA fragment amplified from pRS415 [56] with the MSH6GU (TTTAATTGGAGCAACTAGTTAATTTTGACAAAGCCAATTTGAACTCCAAAAGATTGTACTGAG AGTGCAC) and MSH6GD (ACTTTAAAAAAAATAAGTAAAAATCTTACATACATCGTAAATGAA AATACCTGTGCGGTATTTCACACCG) primers and Phusion Polymerase (New England Biolabs) [98°C for 1 minute followed by 30 cycles of (98°C, 10 sec.; 54°C, 30 sec.; 72°C, 90 sec.)]. We then integrated the *pol2-4* allele into AH0604 using a *URA3*::*pol2-4* chimeric DNA fragment. To generate the *URA3*::*pol2-4* chimeric DNA fragment we first amplified three overlapping DNA fragments. Using the same amplification conditions as above, we amplified *URA3* from pRS416-POL2 [21] with the YIF1KIrp (AGTAAATAGAAAATTTATGACGTAGGAATAAAAGTATATAAGTATTTAACAAATTGGAACAA CACTCAACCCTATCTCGGTCTA) and YIF1KIfp (GAAGAGATCAAAGAGAGGATTTAAT TTCATGCGCATTATTATTATCTACGGTCCAGAGCAGATTGTACTGAGAGTGCACCA) primers, the *POL2* promoter from genomic DNA with the pol2-6376 (GACCGTAGATAATAATAATGCGCATG) and pol2-S3 (CTCAGGAGTTTCCTGGCCTCG) primers, and the *pol2-4* fragment from pRS415pol2-4 [21] with the pol2-seq1F (GGTGGGAGCT TCAAGTCG) and pol2-8752rp (CTCCGGTTTCGGTGTATA CTCAAAGTC) primers. The three fragments were combined in equal-molar ratios and subjected to chimeric PCR [98°C for 1 minute followed by 15 cycles of (98°C, 10 sec.; 72°C, 30 sec.; 58°C, 20 sec.; 72°C, 3 min.)] using YIFKIrp and pol2-8752rp. The entire *POL2* sequence was confirmed by Sanger and Illumina whole genome sequencing.

### Sporulation of AH2801

To isolate haploid mutator mother cells, we first sporulated AH2801 by diluting an overnight culture of the strain 1:50 in YPD and growing the cells until they reached a concentration of 1–2 x 10^7^ cells/ml. The cells were recovered by centrifugation, washed with sterile water, re-suspended in sporulation media (1% potassium acetate, 0.1% yeast extract (Difco), 0.05% Dextrose) at a concentration of 1.5–3 x 10^7^ cell/ml, and then grown for four days at 30°C with shaking. For tetrad dissection, 50 μl of sporulated culture were spun down and re-suspended in 1 M sorbitol with 5 μls of Zymolyase 20T (25μg/μl) (MP Biomedicals) and then incubated for 10 minutes at 30°C to digest the asci walls. Ice-cold sterile water (0.8 ml) was added to suspension and 5 μls were pipetted onto agar plates.

### Fluctuation analysis

For Can^r^ mutation rate measurements, 40 AH2801 tetrads were dissected on SC media. After ~2.5 days of growth, individual colonies (2–3 x 10^6^ cells) were scrapped from the plates and re-suspended in 100 μl of sterile water. 90μl was plated on canavanine-selection plates. The remaining suspension was used for 10-fold serial dilutions, which were plated on SC to determine the total number of cells in each colony (Nt) as well as on canavanine selection plates to accurately count the number of Can^r^ mutants in *pol2-4 msh6*Δ colonies. Since we were blind to the genotypes of the spore clones, at the same time we plated cells on SC-Leu to identify those that carried the *msh6Δ*::*LEU2* allele and on SC-Ura to identify cells carrying *URA3*::*pol2-4*. The AH0401 strain from which AH2801 is derived was designed to facilitate mutation rate measurements in diploids and is heterozygous at the *CAN1* locus (*CAN1*::*natMX/can1Δ*::*HIS3*) [53]. Thus, we also assessed the ability of AH2801 spore clones to grow on SC-His. Clones unable to grow on SC-His carried the *CAN1*::*natMX* allele and were sensitive to canavanine. Mutation counts from these clones were used for mutation rate calculations. After 3 days of growth, colonies on SC and canavanine selection plates were counted and the data grouped according to genotype. Each spore clone was treated as an independent replica culture for fluctuation analyses [27]. Mutation rates were calculated from the mutant counts in each replica culture by estimating the likely number of mutational events (m) by maximum likelihood using newtonLDplating in Salvador 2.3 with Mathematica 8.0 and then divided by the average number of cell divisions inferred from the Nt counts [33,34,57]. Confidence intervals (95%) were calculated with CILDplating in Salvador 2.3.

We estimated the per-base-pair mutation rate from the Can^r^ mutation rate of *pol2-4 msh6Δ* cells using the approach of Drake [35,58,59] as previously described [20].

### Isolation of lineages

In all lineage experiments, cells were grown during the day and stored overnight at 4°C as described [60]. During all incubation steps the plates were wrapped in parafilm. In the first experiments (Lineages A, C, F, G1, G2, and H), tetrads were dissected on non-selective SC media in order to monitor fixation of mutations in WT and *msh6Δ* control cells as well as *pol2-4 msh6Δ* mother cells. One tetrad was dissected per plate and two spores were chosen at random for lineage analysis. Genotyping assays for pol2-4 and msh6Δ alleles were performed as described [21] and verified during whole genome sequencing analysis. During the lineage isolation, mother cells were incubated at 30°C and examined every two hours using a Zeiss Axioskop 40 Tetrad Dissection microscope fitted with a 50μm fiber optic needle. After each cell division, the mother and daughter cells were manually separated using the micromanipulator as previously described [54]. The daughter cells, distinguished by a smaller diameter, were moved to a defined coordinate on the agar plate. In later experiments (Lineages B, D, and E), we focused solely on double mutant spores by dissecting tetrads on media lacking leucine (to select for *msh6Δ*::*LEU2*) and uracil (to select for *URA3*::*pol2-4*). Several tetrads were dissected per plate and a single double mutant spore per plate was selected for analysis by its ability to divide in the absence of leucine and uracil. As before, all daughter cells were moved to a defined coordinate on the agar surface. In addition, the first granddaughter cell born to each daughter cell was also moved to a defined coordinate to serve as a back-up in case the daughter cell died. Dissection continued until double mutant mother cells ceased dividing, whereupon all daughter and granddaughter clones were allowed to form colonies.

### Genome sequencing, data processing and normalization

For sequencing, the entire daughter colony was used to inoculate overnight YPD cultures. Glycerol stocks of each daughter culture were archived and genomic DNA was purified with a ZR Fungal/bacterial purification kit (Zymo Research). The purified DNA was simultaneously fragmented and ligated to Illumina DNA adapters using the Nextera V2 Kit (Illumina), post-indexed by PCR (primer sequences available upon request), and sequenced using 101 bp, paired-end reads on an Illumina 2500 platform. Once all members of a lineage had been sequenced, we used the Burrows-Wheeler Aligner (v0.6.2) [61] to align the reads against a copy of the S288C S. cerevisiae genome (Assembly R64-1-1) in which low complexity and highly repetitive sequences have been removed (<0.5% of the genome) with RepeatMasker. After the initial alignment, unmapped and ambiguously mapped reads were filtered out. PCR duplicates were evaluated using the MarkDuplicates option in the Picard suite of programs (http://picard.sourceforge.net). To further reduce false variant calls, the Genome Analysis Tool Kit (GATK) suite of programs was used for local realignment and base quality score recalibration [62]. We used VarScan2 [63] for variant calling with a minimum read depth of 15, a minimum variant frequency of 0.8, and a quality score of 15. We then filtered the resulting variants to remove strain-specific single nucleotide polymorphisms segregating in our genetic background using a database of putative SNPs segregating in the BY4743 strain background that we previously compiled [53]. We normalized coverage to ensure that we only scored mutations in sequences shared by all members of a lineage. Finally, to determine maternal and daughter specific variants, we compared the mutations found in the last daughter to those found in all preceding daughters. Variants found in the last daughter and shared by one or more of the preceding daughters were designated maternally fixed mutations (See S1 Dataset). Those not found in the maternal lineage were designated daughter-specific mutations and were not evaluated further for this study. Called mutations were then visually confirmed using the Integrated Genome Browser.

### Poisson distributions and finite mixture modeling

Models of expected mutation count data from different Poisson distributions were calculated in Excel. We also used the finite mixture modeling (FMM) procedure of the SAS system (v. 9.4) to compute and compare single-distribution and distribution-mixture models of the mutation count data. A Poisson response distribution and log link function were specified, and parameter estimation was by maximum likelihood. To investigate whether the data could be described better by a negative binomial distribution than by a mixture of two Poisson distributions, we compared the fit of a single negative binomial distribution against the fit of the two-Poisson mixture. Because this comparison cannot be treated as a nested set of hypotheses, we used information theory to characterize the fit, choosing Akaike’s Information Criterion (AIC) for assessment. We again used the FMM procedure of the SAS system (v. 9.4), specifying a Poisson or negative binomial response distribution, and a log link function, for model fitting. Quantification of relative likelihood was after Burnham and Anderson (2002).

### Simulation of the frequency of multiple mutations on the same chromosome

We used 10,000 iterations of a simulation to determine the likelihood that multiple mutations would occur on the same chromosome. To simulate the random distribution of 237 mutations over 87 cell divisions, we generated a sequence file of 87 yeast genomes—each chromosome with a unique identifier. In each iteration of the simulation we randomly selected 237 bases within these 87 yeast genomes and then counted the frequency at which the same chromosome was “mutated” two or three times. From the resulting histogram, we determined the 95% confidence intervals.

### Mutations and replication timing

The locations of all mutations were mapped onto the replication profiles from Raghuraman et al. [39]. The positions of all putative replication origins and termination zones in the replication profiles were identified by noting chromosomal positions where t_rep_ values were at a local maxima or minima. Segments between adjacent maxima and minima were used to define probabilistic replicons to which all mutations were then assigned. The fractional distance of a mutation between any origin/termination pair was calculated by dividing the distance of a mutation to the closest origin by the total distance between the origin and termination zone. The resulting fractional distances were then grouped into bins corresponding to fractional distances of 0.1. To test the significance of the observed distribution, we assumed that random mutagenesis would produce bins of equal size (23.7 mutations/bin). We then compared the observed and expected distributions using a Chi-Squared test. We also performed a similar analysis using a different way of defining replicons outlined by Lujan et al. [50]. In this approach, a replicon is defined as the DNA segment between a confirmed origin (see OriDB, [64]) and halfway to the adjacent confirmed origin (inter-origin midpoint). After assigning mutations to these DNA segments, we then determined the fractional distance of each mutation to the closest origin by dividing the distance of the mutation to the origin by the total distance between the origin and the inter-origin mid-point. As above, we binned mutations by their fractional distance to the origin and determined the significance of the distribution using a Chi-Squared test.

## Supporting Information

S1 Table Summary of mutation accumulation in maternal lineages. (DOCX)

S2 Table Mutation spectra from *pol2-4 msh6*Δ cells. (DOCX)

S3 Table Mutation pairs that co-occur in the same chromosome and cell division. (DOCX)

S1 Fig Can^r^ mutation rates, sample lineage plate, and chromosomal mutation rates. (A) Mutation rates of haploid spore clones determined by fluctuation analysis of canavanine resistance (Can^r^) mutants. *2–4*, *pol2-4*; *m6*, *msh6Δ*; error bars, 95% confidence intervals. (B) Representative pedigrees for single cell mutation rates. Two spores from the same tetrad were selected for each lineage. One spore was *pol2-4 msh6*Δ and divided 16 times (yellow *d12*, *d13*, and *d16* indicate locations of daughters). The other spore was *POL2 MSH6* and divided 24 times (orange *d12*, *d13*, and *d24* indicate locations of daughters). (C) Mutation rate of all 16 chromosomes determined by dividing the number of mutations for each chromosome by the chromosome size.
(EPS)

S2 Fig Poisson models. (A) Observed and modeled Poisson distributions for each lineage. The observed distribution of *k* mutation counts (blue bars, Data) for each lineage is plotted alongside the predicted distribution of *k* mutation counts determined with a single Poisson Model (red bars, Poisson Model) calculated using the average mutation rate of 2.6 x 10^-7^ mutations/bp/cell division, the size of the sequenced genome of each lineage, and the number of cell divisions scored in each lineage (see S1 Dataset). (B) Combined lineage data and model. The observed and predicted distributions of *k* mutation counts from each lineage were summed to produce combined distributions of the data (Combined Data) and predicted mutation counts (Summed Poisson Model). (C) The Summed Poisson Model was compared to a less complicated Poisson Model (Simplified Poisson model), which utilized the average mutation rate, the average genome size (1.02 x 10^7^ base-pairs), and the total number of scorable cell divisions across all lineages (85, *note that 2 cell divisions in lineage F were not scorable*).
(EPS)

S3 Fig Correlation test for zero-inflation. Data that are undercounted have too many observations with zero events (zero-inflation). If undercounting was a problem in our data, smaller lineages would have a disproportionately high number of cell divisions with 0 mutations. The plot shows the number of divisions with zero mutations in each lineage versus the size of the sequenced genome. No correlation is observed.
(EPS)

S4 Fig Locations of mutations within the replication profiles of all 16 yeast chromosomes. Chromosome number is given to the right of each trace. peaks, origins of replication; valleys, termination zones where replication forks from adjacent origins collide; darker green triangles, mutations that occurred by themselves on the given chromosome in distinct cell divisions; light green triangles and all other colors, mutation pairs or trios that co-occurred in the same cell division; red circles, centromeres.
(EPS)

S5 Fig Correlation tests related to the co-occurrence of mutations on the same chromosome in the same cell division. All Pearson correlation values (r) were calculated in Microsoft Excel assuming a linear correlation. (A) Chromosomal Position. The chromosomal locations of each mutation in a pair (relative to the centromere) serve as X and Y coordinates of each co-occurrence. (B) Replication timing. The predicted replication timing values (t_rep_) (see Raghuraman et al. [39]) of each mutation in a pair serve as the X and Y coordinates of each co-occurrence.
(EPS)

S6 Fig Distribution of mutations relative to origins and the inter-origin midpoints. *Top*. Histogram of the fractional distances of mutations to the nearest origin. *Bottom*. Schematic shows how fractional distances are determined. Replicons were defined as the distance between an origin and halfway to the adjacent origin (inter-origin midpoint).
(EPS)

S1 Dataset Cell lineage data. The data for each lineage are found in separate tabs. The variant calls for each cell division are tabulated in the upper table. The genomic coordinates for each mutation are reported as the NCBI reference number and position of the mutated base, which are separated by a colon. New variants in each daughter clone are highlighted in orange. Mutations co-occurring in the same chromosome and cell division are outlined with a black box.
(XLSX)

S2 Dataset Poisson distribution calculations. We show the tables used to model the experimental data to either a single- or double-Poisson models. The top table summarizes the results found in Lineages B-H in S1 Dataset. The second table reports the predicted number of cell divisions with *k* number of mutations using a single-Poisson as a model. Three different mutation rates are tabulated (0.4x10^-7^, 2.6x10^-7^, and 4x10^-7^). The third set of tables compares the actual data to the summed Poisson models from each lineage (See S1 Dataset) and simplified Poisson models. These data were used in the production of Fig 2, and S2 Fig’
(XLSX)

S3 Dataset Fractional distances of mutations to origins and termination zones. We show both the physical and fractional distances of all reported mutations to the closest origins and termination zones, as defined by Raghuraman et al [39]. The fractional distance was calculated as described in the Materials and Methods. Data are sorted by fractional distance from the origin to the nearest termination zone and grouped into bins corresponding to fractional distances of 0.1. The counts from each bin were used in making Fig 4.
(XLSX)
