# Supplementary material for: Volatility of Mutator Phenotypes at Single Cell Resolution
Source: PLoS Genet. 2015 Apr 13;11(4):e1005151. doi: 10.1371/journal.pgen.1005151 (PMC4395103; doi:10.1371/journal.pgen.1005151)
Supplement: S1 Table — (DOCX) [file pgen.1005151.s001.docx]

**S1 Table:** Summary of mutation accumulation in maternal lineages.

| **Sample**  **ID** | **Cell**  **Division** | **# New**  **Mutations** | **Sequenced**  **Bases** | **Mutation**  **Rate** |
| --- | --- | --- | --- | --- |
| **Lineage A** | | | | |
| **6_1_3** | 3 | 1 |  |  |
| **6_1_4** | 4 | 0 |  |  |
| **6_1_5** | 5 | 0 |  |  |
| **6_1_6** | 6 | 0 |  |  |
| **6_1_7** | 7 | 0 |  |  |
| **6_1_8** | 8 | 1 |  |  |
| **6_1_9** | 9 | 0 |  |  |
|  |  |  |  |  |
| **Lineage B** | | | | |
| **5_5** | 3 | 0 | 10926041 | <9.2E-08 |
| **5_7** | 4 | 2 | 10926041 | 1.8E-07 |
| **5_9** | 5 | 0 | 10926041 | <9.2E-08 |
| **5_11** | 6 | 3 | 10926041 | 2.7E-07 |
| **5_13** | 7 | 2 | 10926041 | 1.8E-07 |
| **5_15** | 8 | 4 | 10926041 | 3.7E-07 |
| **5_17** | 9 | 1 | 10926041 | 9.2E-08 |
| **5_19** | 10 | 3 | 10926041 | 2.7E-07 |
|  |  |  |  |  |
| **Lineage C** | | | | |
| **5_1_3** | 3 | 3 | 10034503 | 3.0E-07 |
| **5_1_4** | 4 | 7 | 10034503 | 7.0E-07 |
| **5_1_5** | 5 | 6 | 10034503 | 6.0E-07 |
| **5_1_6** | 6 | 0 | 10034503 | <1.0E-07 |
| **5_1_7** | 7 | 0 | 10034503 | <1.0E-07 |
| **5_1_8** | 8 | 2 | 10034503 | 2.0E-07 |
| **5_1_9** | 9 | 3 | 10034503 | 3.0E-07 |
| **5_1_10** | 10 | 4 | 10034503 | 4.0E-07 |
| **5_1_11** | 11 | 4 | 10034503 | 4.0E-07 |
| **5_1_12** | 12 | 6 | 10034503 | 6.0E-07 |
| **5_1_13** | 13 | 5 | 10034503 | 5.0E-07 |
| **5_1_14** | 14 | 1 | 10034503 | 1.0E-07 |
| **5_1_15** | 15 | 0 | 10034503 | <1.0E-07 |
|  |  |  |  |  |
| **Lineage D** | | | | |
| **8_5** | 3 | 2 | 10099122 | 2.0E-07 |
| **8_7** | 4 | 2 | 10099122 | 2.0E-07 |
| **8_9** | 5 | 0 | 10099122 | <9.9E-08 |
| **8_11** | 6 | 3 | 10099122 | 3.0E-07 |
| **8_13** | 7 | 0 | 10099122 | <9.9E-08 |
| **8_15** | 8 | 0 | 10099122 | <9.9E-08 |
| **8_17** | 9 | 1 | 10099122 | 9.9E-08 |
| **8_19** | 10 | 1 | 10099122 | 9.9E-08 |
| **8_22^*^** | 11 | 4 | 10099122 | 4.0E-07 |
| **8_23** | 12 | 5 | 10099122 | 5.0E-07 |
| **8_25** | 13 | 3 | 10099122 | 3.0E-07 |
| **8_27** | 14 | 0 | 10099122 | <9.9E-08 |
| **8_30^**^** | 15 | 9 | 10099122 | 8.9E-07 |
| **8_31** | 16 | 4 | 10099122 | 4.0E-07 |
| **8_33** | 17 | 2 | 10099122 | 2.0E-07 |
|  |  |  |  |  |
| **Lineage E** | | | | |
| **9_5** | 3 | 2 | 10889177 | 1.8E-07 |
| **9_7** | 4 | 3 | 10889177 | 2.8E-07 |
| **9_9** | 5 | 1 | 10889177 | 9.2E-08 |
| **9_11** | 6 | 6 | 10889177 | 5.5E-07 |
| **9_13** | 7 | 0 | 10889177 | <9.2E-08 |
| **9_15** | 8 | 0 | 10889177 | <9.2E-08 |
| **9_17** | 9 | 0 | 10889177 | <9.2E-08 |
| **9_19** | 10 | 8 | 10889177 | 7.3E-07 |
| **9_21** | 11 | 0 | 10889177 | <9.2E-08 |
| **9_23** | 12 | 10 | 10889177 | 9.2E-07 |
|  |  |  |  |  |
| **S1 Table: *continued*** | |  |  |  |
| **Sample**  **ID** | **Cell**  **Division** | **# New**  **Mutations** | **Sequenced**  **Bases** | **Mutation**  **Rate** |
| **Lineage F** | | | | |
| **9_1_3** | 3 | 3 | 11040018 | 2.7E-07 |
| **9_1_4** | 4 | 7 | 11040018 | 6.3E-07 |
| **9_1_5** | 5 | 1 | 11040018 | 9.1E-08 |
| **9_1_6** | 6 | 3 | 11040018 | 2.7E-07 |
| **9_1_7** | 7 | 2 | 11040018 | 1.8E-07 |
| **9_1_8^†^** | 8 | - | - | - |
| **9_1_9^†^** | 9 | - | - | - |
| **9_1_10** | 10 | 0 | 11040018 | <9.1E-08 |
| **9_1_11** | 11 | 5 | 11040018 | 4.5E-07 |
| **9_1_12** | 12 | 2 | 11040018 | 1.8E-07 |
| **9_1_13** | 13 | 3 | 11040018 | 2.7E-07 |
| **9_1_14** | 14 | 1 | 11040018 | 9.1E-08 |
|  |  |  |  |  |
| **Lineage G1** | | | | |
| **12_1_3** | 3 | 1 | 9684038 | 1.0E-07 |
| **12_1_4** | 4 | 1 | 9684038 | 1.0E-07 |
| **12_1_5** | 5 | 0 | 9684038 | <1.0E-07 |
| **12_1_6** | 6 | 0 | 9684038 | <1.0E-07 |
| **12_1_8** | 7 | 3 | 9684038 | 3.1E-07 |
| **12_1_10** | 8 | 6 | 9684038 | 6.2E-07 |
| **12_1_12** | 9 | 4 | 9684038 | 4.1E-07 |
| **12_1_14** | 10 | 5 | 9684038 | 5.2E-07 |
| **12_1_19** | 11 | 3 | 9684038 | 3.1E-07 |
| **12_1_20** | 12 | 5 | 9684038 | 5.2E-07 |
| **12_1_21** | 13 | 4 | 9684038 | 4.1E-07 |
|  |  |  |  |  |
| **Lineage G2** | | | | |
| **12_1_9** | 5 | 7 | 10209723 | 6.9E-07 |
| **12_1_11** | 6 | 6 | 10209723 | 5.9E-07 |
| **12_1_13** | 7 | 0 | 10209723 | <9.8E-08 |
| **12_1_15** | 8 | 5 | 10209723 | 4.9E-07 |
| **12_1_16** | 9 | 0 | 10209723 | <9.8E-08 |
|  |  |  |  |  |
| **Lineage H** | | | | |
| **14_2_3** | 3 | 7 | 10826564 | 6.5E-07 |
| **14_2_4** | 4 | 0 | 10826564 | <9.2E-08 |
| **14_2_5** | 5 | 2 | 10826564 | 1.8E-07 |
| **14_2_6** | 6 | 0 | 10826564 | <9.2E-08 |
| **14_2_7** | 7 | 4 | 10826564 | 3.7E-07 |
| **14_2_8** | 8 | 3 | 10826564 | 2.8E-07 |
| **14_2_9** | 9 | 4 | 10826564 | 3.7E-07 |
| **14_2_10** | 10 | 1 | 10826564 | 9.2E-08 |
| **14_2_11** | 11 | 4 | 10826564 | 3.7E-07 |
| **14_2_12** | 12 | 1 | 10826564 | 9.2E-08 |
| **14_2_13** | 13 | 1 | 10826564 | 9.2E-08 |
| **14_2_14** | 14 | 3 | 10826564 | 2.8E-07 |
