# Supplementary material for: Volatility of Mutator Phenotypes at Single Cell Resolution
Source: PLoS Genet. 2015 Apr 13;11(4):e1005151. doi: 10.1371/journal.pgen.1005151 (PMC4395103; doi:10.1371/journal.pgen.1005151)
Supplement: S2 Table — (DOCX) [file pgen.1005151.s002.docx]

|  | **G→A** | **G→T** | **C→A** | **C→T** | **T→C** | **A→C** | **A→G** | **T→A** | **T→G** | **A→T** |
| --- | --- | --- | --- | --- | --- | --- | --- | --- | --- | --- |
| **Lineage B** | 1 | 9 | 5 | 5 | 0 | 0 | 0 | 0 | 0 | 0 |
| **Lineage C** | 11 | 6 | 11 | 9 | 1 | 1 | 2 | 0 | 0 | 0 |
| **Lineage D** | 6 | 10 | 7 | 7 | 0 | 0 | 4 | 1 | 0 | 0 |
| **Lineage E** | 8 | 9 | 5 | 0 | 3 | 2 | 0 | 0 | 1 | 0 |
| **Lineage F** | 4 | 7 | 6 | 3 | 6 | 2 | 2 | 0 | 0 | 0 |
| **Lineage G1** | 6 | 3 | 13 | 4 | 1 | 0 | 3 | 2 | 0 | 1 |
| **Lineage G2** | 0 | 3 | 8 | 3 | 4 | 0 | 1 | 1 | 0 | 1 |
| **Lineage H** | 5 | 0 | 10 | 2 | 2 | 3 | 4 | 0 | 2 | 0 |
| **Total** | 41 | 47 | 65 | 33 | 17 | 8 | 16 | 4 | 3 | 2 |

**S2 Table:** Mutation spectra from *pol2-4 msh6*Δ cells.
