# Supplementary material for: Volatility of Mutator Phenotypes at Single Cell Resolution
Source: PLoS Genet. 2015 Apr 13;11(4):e1005151. doi: 10.1371/journal.pgen.1005151 (PMC4395103; doi:10.1371/journal.pgen.1005151)
Supplement: S3 Table — (DOCX) [file pgen.1005151.s003.docx]

| **Sample** | **Chromosome ID** | **Position of Mutations (relative to CEN)** | | **Replication Timing^a^** | |
| --- | --- | --- | --- | --- | --- |
| **Lineage B, Division 8** | NC_001139 | 406691 | 541667 | 27.8 | 42.6 |
| **Lineage C, Division 4** | NC_001145 | -184614 | -152368 | 29.1 | 28.6 |
| **Lineage C, Division 5** | NC_001144 | 181937 | 158610 | 38.9 | 33.3 |
| **Lineage C, Division 9** | NC_001144 | 204449 | 209273 | 25.9 | 22.6 |
| **Lineage C, Division 11** | NC_001139 | 515057 | -469335 | 34.8 | 36.8 |
| **Lineage C, Division 13** | NC_001144 | -36767 | 783848 | 31.1 | 34.0 |
| **Lineage C, Division 14** | NC_001136 | 851305 | 165508 | 42.4 | 34.7 |
| **Lineage C, Division 14** | NC_001143 | -117242 | -36589 | 28.9 | 25.9 |
| **Lineage D, Division 12** | NC_001146 | -532669 | -446627 | 29.1 | 26.6 |
| **Lineage D, Division 15** | NC_001142 | 208591 | -1815 | 24.2 | 29.7 |
| **Lineage D, Division 16** | NC_001144 | 171073 | 668135 | 32.6 | 38.4 |
| **Lineage E, Division 4** | NC_001139 | 94512 | -103308 | 30.5 | 30.6 |
| **Lineage E, Division 6** | NC_001136 | -295194 | 600425 | 36.7 | 47.2 |
| **Lineage E, Division 6** | NC_001147 | -12950 | -184985 | 23.3 | 25.2 |
| **Lineage E, Division 10** | NC_001145 | 229518 | -94566 | 16.6 | 20.2 |
| **Lineage E, Division 12** | NC_001136 | 865529 | 877431 | 42.5 | 34.9 |
| **Lineage E, Division 12** | NC_001139 | 579300 | -454111 | 37.8 | 38.7 |
| **Lineage F, Division 4** | NC_001148 | -424927 | 225921 | 18.2 | 31.3 |
| **Lineage F, Division 6** | NC_001137 | 201101 | 8462 | 22.5 | 24.5 |
| **Lineage F, Division 11** | NC_001146 | -469189 | -128427 | 26.6 | 29.9 |
| **Lineage G1, Division 12** | NC_001136 | -274652 | -310801 | 35.6 | 48.6 |
| **Lineage G1, Division 12** | NC_001147 | 477026 | 725278 | 18.6 | 33.3 |
| **Lineage G2, Division 5** | NC_001147 | 29171 | -41572 | 16.6 | 21.8 |
| **Lineage G2, Division 8** | NC_001136 | 32998 | 145293 | 43.5 | 24.4 |
| **Lineage G2, Division 8** | NC_001139 | 561057 | 45780 | 37.1 | 39.8 |
| **Lineage H, Division 3** | NC_001144 | 549814 | 830998 | 39.2 | 37.4 |
| **Lineage H, Division 8** | NC_001144 | 859355 | -135857 | 40.0 | 28.5 |
| **Lineage H, Division 9** | NC_001148 | -348210 | 124489 | 25.2 | 23.8 |
| **Lineage H, Division 11** | NC_001142 | 232527 | -392784 | 38.3 | 25.9 |
| **Lineage H, Division 14** | NC_001145 | 389125 | 560949 | 24.7 | 23.5 |
| ^a^based on T_rep_ values from Raghuraman et al. | | | |  |  |

**S3 Table:** Mutation pairs that co-occur in the same chromosome and cell division.
